# Comparison of nucleated red blood cells in the umbilical cord of term neonates in healthy women and women with preeclampsia

**Published:** 2013-01

**Authors:** Roya Faraji Darkhaneh, Atefeh Ghanbari, Maryam Asgharnia, Mitra Kian

**Affiliations:** 1 *Reproductive Health Research Center, Department of Obstetrics and Gynecology, Guilan University of Medical Sciences, Guilan, Iran.*; 2 *Department of Nursing Education, Reproductive Health Research Center, Social Determinant of Health Research Center, Guilan University of Medical Sciences, Rasht, Iran.*

## Abstract

**Background: **Asphyxia is a common cause of perinatal mortality in 5-10% of all births worldwide. The present parameters for determining perinatal asphyxia, e.g. preeclampsia, cannot be considered as markers per se, and require auxiliary markers, e.g. increased number of nucleated red blood (NRBC) cells, for early diagnosis of perinatal asphyxia.

**Objective:** In this study, we evaluated the mean NRBC count in preeclampsia and to determine the usefulness of the NRBC as independent prognostic factors of perinatal complications.

**Materials and Methods:** This was a cross-sectional study in order to compare the NRBC in the umbilical cord of term neonates born to 50 mothers with preeclampsia and 150 normal mothers. The exclusion criteria were mother’s affliction with complications of pregnancy and inexact last menstrual period. The variables under study were maternal and neonatal data. The count of NRBC was determined with standard laboratory procedures in the blood samples from umbilical cord of the neonates. The acquired data were fed into SPSS 16 software and analyzed using statistical tests.

**Results:** The mean value of NRBC count was significantly higher in preeclamptic women (p<0.0001). The average 1^st^ and 5^th^ minute Apgar scores were significantly higher in normal mothers (p<0.001).

**Conclusion:** Increase of NRBC in neonates born to mothers with preeclampsia may be due to chronic hypoxia; this group of neonates has increased risk and requires more precise and extensive care during delivery and after birth in order to have reduced mortality and complications during the neonatal period.

## Introduction

Preeclampsia is a syndrome of pregnancy in which organ perfusion is compromised due to vasospasm and endothelial activation. It is marked by proteinuria and hypertension. Proteinuria is defined as urinary excretion of protein more than 300 mg in 24 hours or a constant amount of 30 mg/dL (1+ dipstick). It occurs when the specific glomerular lesion of preeclampsia appears. 

The minimum criteria for preeclampsia are blood pressure ≥140/90 after the 20^th^ week of gestation and proteinuria of over 300 mg/dL in 24 hours or dipstick results of ≥1+ in random samples (1). Numerous studies have indicated that in response to intrauterine hypoxia, such as intrauterine growth restriction (IUGR), maternal diabetes, preeclampsia, prematurity and anemia, the sources of NRBC released into bloodstream increase, thus raising the number of NRBC (2-4). 

The NRBCs, which are in fact premature precursors of the red blood cells, are released from the fetal bone marrow in response to the increased erythropoietin caused by hypoxia. Researchers have mentioned increased NRBC in term neonates as an indicator of chronic intrauterine hypoxia. This rise has been reported to start as early as 2 hours after hypoxia; the longer the duration of asphyxia, the more intense will be the rise in NRBCs (5). 

Asphyxia, the foremost cause of perinatal mortality worldwide, occurs in 5-10% of all births. The parameters used for predicting or defining asphyxia include Apgar score, acidemia of the umbilical cord artery, fetal heart monitoring, patterns of heartbeat, pH metry of the blood of fetal head, increased level of serum erythropoietin, and presence of meconium in amniotic fluid (6). Recently, the count of umbilical cord NRBC for every 100 white blood cells has been introduced as a marker of perinatal asphyxia (7). 

In fact, the hematopoietic system responds to hypoxia with increasing erythropoietin and NRBC (8, 9). Increased counts of fetal NRBC have been reported in hypoxic fetal situations such as IUGR and fetal distress (7). Preeclampsia is a common disorder which alongside hemorrhage and infection comprises a lethal triad, accounting for the majority of pregnancy-related fatalities (1). In a study by Aali *et al* in Kerman, the findings indicated that the average count of NRBC per 100 WBC was significantly higher in the preeclampsia group (18.2±31.8) compared to the normal group (6.2±8.1). 

Their study indicated low birth weight (LBW) and IUGR to be significantly related to the abnormal counts of NRBC in mothers with preeclampsia. Thus, the fetal response to placental-uterine failure in mothers with preeclampsia results in increased counts of NRBC in the blood of umbilical cord, particularly if it accompanies LBW and IUGR, in which reduced perfusion of placenta affects the number of NRBC in the blood of umbilical cord in mothers with preeclampsia (10). 

A study by Gosh *et al* in India on 26 asphyxiated term neonates (group 1) and 49 non-asphyxiated term neonates (group 2) indicated that the neonates in group 1 had 1^st^ minute Apgar scores of less than 6 and arterial pH of less than 7.15, whereas the second group had Apgar scores of more than 6 and arterial pH of more than 7.15. 

The study showed 38.4% of neonates in the first group manifested evidences of ischemic and hypoxic encephalopathy while none of these were observed in the second group. The counts of NRBC for the first and the second group were 16.5±6.4 and 8.6±7.01, respectively, which were significantly different (p<0.001). The amount of hemoglobin and NRBC count were significantly higher in the first group. The NRBC count per 100 WBC was inversely related to the Apgar score and umbilical artery pH (p<0.001 and r=-0.5 for Apgar score; p<0.001 and r=-0.048 for pH). 

In neonates of the second group, the NRBC count was significantly related to asphyxia of birth, hypertension of pregnancy and IUGR. Therefore, the level of NRBC is related to acute and chronic asphyxia and thus NRBC may be used as an index for evaluations of the neonatal period (7). In a similar study in Turkey, 69 high risk neonates (including 14 cases of IUGR, 25 premature neonates, 18 asphyxiated term neonates and 12 neonates born to diabetic mothers) were compared to 37 healthy term neonates (18 cases of normal vaginal delivery and 19 cases of Caesarean section) to indicate that the NRBC count was not significantly difference among the high risk groups, whereas it was significantly higher in the high risk groups compared to the control group. 

Logarithm of NRBC in neonates with good prognosis was significantly lower compared to that of neonates with poor prognosis (4). Since the relationship between preeclampsia and intrauterine hypoxia with NRBC and its accompanying factors have not been established yet, we decided to use NRBC count as one of the indices of asphyxia to compare in neonates born to mothers with preeclampsia and those born to healthy mothers. In order to provide the optimal care for term babies born to mothers with preeclampsia. 

## Materials and methods

This study was done with financial support of Guilan University of Medical Sciences. This is a cross-sectional study comparing the count of NRBCs in the blood of the umbilical cord of healthy term neonates with term neonates born to mothers with preeclampsia in Alzahra Hospital, Rasht in 2009. The study was approved by the ethics committee of Guilan University of Medical Science and informed consent was obtained from each subject. 

The inclusion criteria were all term pregnant women that were admitted in Alzahra Hospital for delivery in 2009. In total 50 mothers diagnosed with preeclampsia (BP≥140/90 and proteinuria of over 300 mg/dL in 24 hours or dipstick results of ≥1+ in random samples) at gestational ages of over 37 weeks and 150 healthy term mothers entered the study. The exclusion criteria were mother’s affliction with complications of pregnancy such as diabetes, hypertension without proteinuria, chorioamionitis, cigarette smoking, LBW of neonates (less than 2500 grams), twin birth or neonates with cyanotic heart disease. 

In addition, mothers with inexact last menstrual period (LMP) were excluded from the study. The variables under study were the obstetric data recorded by a resident of gynecology, including maternal age, number of parity, gestational age in weeks and days from the LMP. Neonatal data including gender, weight at birth in grams, level of hemoglobin, count of white blood cells and NRBCs in the blood of umbilical cord at birth, outcome of neonate based on 1^st^ and 5^th^ minute Apgar scores (with Apgar scores of 7-10 considered normal), neonatal problems reported by a specialist or resident of neonatology in the first 48 hours of birth, such as convulsion, jaundice, hypotonia, sepsis, admission to neonatal intensive care unit, and their status of discharge or hospitalization.

In order to evaluate the hematologic factors, 2CC of the arterial blood of the umbilical cord was aspirated with a syringe immediately after birth, deposited in tubes containing ethylene di-amine tetra acetic (EDTA) and sent to the laboratory of the hospital for hematologic analysis. Using an automatic hematologic blood cell counter, the number of WBC/mm^3^ and the level of hemoglobin (g/dL) were determined. Subsequently, blood smears on a glass slide were prepared and stained with the Wright method; then the number of NRBCs (NRBC per 100 WBC) was determined manually by one person only-the laboratory physician. Furthermore, the count of NRBC was determined using the percentage of NRBC and the number of WBC per cubic milliliter blood of umbilical cord. 

**Statistical analysis**

Data were analyzed using SPSS version16. The normality of continuous data was checked by One Sample Kolmogorov-Smirnov Test, and between G groups comparison was done by a one Chi-square, Student’s *t*-test and one way ANOVA. The statistical significant was set at 0.05 level.

## Results

Analysis of the demographic data of the samples indicated that the mean age of mothers in our study was 27.54±4.66 years, randeg from 18-40 years of age. There was no statistically significant difference between the mean age of mothers in the preeclampsia group (27.48±5.07 years) and that of mothers on the normal group (27.56±4.53 years). 

The average number of gravidity was 1.71±0.86 (minimum 1 and maximum 4) for mothers in our study. The mean number of gravidity for mothers was 1.74±0.96 in the preeclampsia group and 1.7±0.83 in the normal group, indicating no significant difference. The mean gestational age was 38.64±1.06 weeks (minimum 37 weeks and maximum 41 weeks). The mean gestational age for mothers with preeclampsia was 37.48±0.83 weeks which is significantly lower compared to the normal mothers with a mean gestational age of 39.02±0.82 weeks (p<0.001). 

Overall 40% (20 cases) of mothers with preeclampsia had normal delivery and 60% (30 cases) had caesarean section. The normal mothers had normal delivery and caesarean section in 48.7% (73 cases) and 51.3% (77 cases), respectively, which indicates no significant difference from the preeclampsia group. The newborns’ gender was 60% (30 cases) male and 40% (20 cases) female in the preeclampsia group. In the normal group, 53.3% (80 cases) were boys and 46.7% (70 cases) were girls, indicating no significant difference between the two groups. 

The mean weight at birth was 3108.87±426.16 g, with a maximum of 4000 g and minimums of 2030 g. The mean weights of neonates born to mothers with preeclampsia and normal mothers were 2795.8±558.8 g and 3213.2±309.85 g, respectively, indicating a statistically significant difference. The mean level of umbilical hemoglobin was 14.09±1.69. 

The level of umbilical hemoglobin was not significantly different between the two groups. The mean count of NRBC was 618.31±725.01 for both groups. Analysis of the NRBC count in either group indicated the umbilical NRBC count to be significantly higher in the preeclampsia group compared to the normal group (1430.16±825.9 vs. 347.60±427.5; p=0.0001) (Table I).

As for the normal level of NRBC (normal below 10%, abnormal increased equal to or greater than 10%), our findings indicate that in 64% (32 cases) of mothers with preeclampsia and 3.3% (5 cases) of normal mothers, an abnormal level of NRBC was detected; the two groups were significantly different in this regard (p<0.0001) (Table II). Table III compares the mean percentage of NRBCs in the umbilical cord of term neonates born to mothers with preeclampsia and normal mothers, indicating a significant difference between the two groups in terms of the mean percentage of NRBC.

Comparing the groups in terms of neonatal outcome (neonatal Apgar score, status of admission and discharge) indicated the 1^st^ minute Apgar score of the normal group (mean 8.44±0.6) to be significantly greater than the 1^st^ minute Apgar score of the preeclampsia group (mean 8.08±1.12; p<0.001). Furthermore, the 5^th^ minute Apgar score was significantly different between the two groups (9.42±0.61 vs. 9.12±0.89; p<0.001). Comparing the status of the newborns for complications such as pulmonary infection, sepsis and jaundice yielded no significant difference between the two groups. We observed no statistically significant difference between the NRBC counts per 100 WBC of term neonates born in both groups who developed jaundice (p=0.044). 

Furthermore, no significant difference was observed between the NRBC counts per 100 WBC of term neonates born in both groups who developed pulmonary infection (p=0.061). Data analysis revealed no significant difference between the mean NRBC count and type of delivery, Apgar scores of 6 or lower. 

However, a significant difference was observed between the NRBC counts of the preeclampsia and the normal groups in neonates with 1^st^ minute Apgar scores of 7 or more (p=0.0001). Stepwise regression indicated that the variables of gestational week on delivery, type of delivery, neonatal outcome, age, 1^st^ minute Apgar score, and WBC count influence the mean percentage of NRBCs and these variables create 64% variance in the mean percentage of NRBCs.

**Table I:** Comparison of mean of Hb, WBC and NRBC/100WBC in the two groups

**Group**	**Control group (n=150)**	**Preeclampsia group (n=50)**	p-value*
Hb (Mean±SD)	14.12 ± 1.71	14 ± 1.66	0.659
Corrected cord blood WBC (Mean±SD)	11934.6 ± 2672.7	12584.2 ± 2507	0.132
NRBC per100 WBC (Mean±SD)	347.69 ± 427.5	1430.16 ± 825.9	0.0001

* Student’s *t*-test

**Table II:** Distribution of frequency of normal levels of NRBC/100WBC in the two groups

**Group**		**Preeclampsia group (n=50)** **Number (%)**	**Control group (n=150)** **Number (%)**	p-value*
NRBC Normality				
	Normal <10%	18 (36)	145 (96.7)	0.0001
	Abnormal >10%	32 (64)	5 (3.3)	

* chi-square

NRBC: Nucleated red blood cells

**Table III:** Comparison the mean percentage of nucleated red blood cells (NRBC) in two groups

**Group**	**Nucleated red blood cells (Mean± SD)**	p-value*
Preeclampsia group	11.12 (5.5)	0.0001
Normal group	2.74 (2.9)	

* Student’s *t*-test

## Discussion

The mean weight of neonate in the present study was 3108.87±426.16 g. In the preeclampsia group, the mean weight was 2795.8±558.8 g and in the normal group, it was 3213.2±309.85 g which is significantly higher than the preeclampsia group; a finding in line with those of Ali *et al* who reported mean weight of neonates to be 2663.7±726.7 g in the preeclampsia group and 3208.3±437.5 g in the normal group (p<0.05) (12). 

In a study by Gosh *et al* no statistically significant difference was observed between the birth weights of neonates in the asphyxia and non-asphyxia group (7). Furthermore, Saracoglo *et al* reported no significant difference between the birth weights of the acute fetal distress group and the normal group, while the birth weight of neonates with chronic fetal distress was significantly lower compared to the control group (11).

One of the limitations of our study was due to fewer patients in preeclamptic group and another was that our attempt to match the patients for gestational age between two groups wasn’t possible. In the present study, the umbilical WBC count was not significantly different between the two groups; a finding which is consistent with findings of Ali *et al* and Gosh *et al* (7, 10). In the Saracoglo *et al* study, the mean count of WBC in the umbilical cord was not significantly different between the acute respiratory distress and control groups, while it was significantly higher in the chronic respiratory distress group (11).

In our study, the NRBC count per mm^3^ of umbilical cord blood of the newborn was significantly higher in the preeclampsia group (1430.16±825.9) compared to the normal group (347.69±427.5). In the present study, the mean NRBC count per 100 WBC in the umbilical cord was 11.12±5.5 for newborns in the preeclampsia group and 2.74±2.9 for newborns in the normal group (p<0.05). In the Bibi Shahnaz Aali *et al* study, the mean NRBC count per 100 WBC in the umbilical cord was 18.2±31.8 in newborns of the preeclampsia group and 6.2±8.1 in newborns of the normal group, indicating a significant difference (10). 

Saracoglo *et al* reported a significant difference between the mean NRBC count per 100 WBC in chronic fetal distress group (24.43±20.05) and acute fetal distress (11.18±4.29) and control groups (7.56±3.85) (11). Similarly, Gosh *et al* observed that the mean NRBC count per 100 WBC is significantly higher in neonates with asphyxia (16.5±6.4) compared to those without asphyxia (8.6±7.1) (7).

In this study, the newborns of mothers with preeclampsia and normal mothers were not significantly different in terms of admission and discharge. The NRBC count in neonates who were discharged without any problem was significantly higher in those who were born to mothers with preeclampsia compared to the control group. Moreover, the NRBC count of neonates who developed icterus was significantly higher in those born to mothers with preeclampsia compared to those born to normal mothers. In a study by Hanlon-Londberg *et al*, neonates with increased NRBC were more likely to be admitted to the neonatal intensive care unit (3). In the Gosh *et al* study, the rate of admission to the NICU was significantly higher for the case group than the control group (7).

Preeclampsia is a common disorder, forming a fatal triad alongside hemorrhage and infection (1). In preeclampsia, placental perfusion is compromised due to vasospasm, increasing the prenatal fatality and complications. Reduced placental-uterine perfusion in preeclampsia results in decreased perfusion of the fetus, which in turn leads to fetal growth restriction and chronic hypoxia (13). Moreover, as mentioned earlier, the hematopoietic system responds by increasing erythropoietin and the subsequent release of premature forms of red blood cells into the bloodstream within hours to days from the onset of hypoxia (14, 15). Our study indicates the total count of NRBC per mm^3^, as well as the NRBC count per 100 WBC, to be higher in neonates born to mothers with preeclampsia compared to those born to normal mothers.

Various studies have indicated that the mean total NRBC count per mm^3^ is about 500 in healthy, term neonates and values greater than 1000 per mm^3^ may be considered as elevated. In addition, the NRBC/100WBC ratio from 0-10 is normal, and values higher than 10-20 NRBC/100WBC are considered elevated. Nevertheless, the total count of blood leukocytes affects this ratio, as well (8).

According to previous studies, the elevated count of NRBCs in term neonates is related to hypoxemia, acidemia, asphyxia, lower Apgar scores, increased admission to NICU and increased neurologic injuries caused by asphyxia (6, 9, 16-18). However, due to the extensive overlap observed among cases of elevated NRBC count in acute, subacute, and chronic asphyxia and even without asphyxia, (19, 20), the NRBC count or maternal preeclampsia cannot be used definitively as an indicator of intra-uterine asphyxia (8, 16). 

Nonetheless, increased NRBC alongside maternal preeclampsia may indicate chronic hypoxia starting a few days prior to delivery (10). Thus, these neonates are at an increased risk for neonatal complications of chronic hypoxia such as gastrointestinal bleeding, ischemic necrosis of myocardium, hypocalcemia and hypoglycemia, RDS, cerebral hemorrhage and renal cortical necrosis (15) and must be given extra care. Considering the fact that NRBC count increases in neonates born to mothers with preeclampsia, and this increase may be due to chronic hypoxia, these neonates are in the high risk group and require more precise evaluations and greater care during delivery and after birth in order to reduce their complications and fatality during the neonatal period. 

Therefore, we recommend future studies to be conducted in a prospective manner to determine the range of elevated NRBC count, as well as determining its sensitivity and specificity so as to provide the possibility of predicting the short term or long term complications of neonates born to mothers with preeclampsia. 
